# Psychological and physical factors related to social integration of older adults in Ghana

**DOI:** 10.1186/s12877-024-04954-x

**Published:** 2024-04-23

**Authors:** Joseph Kojo Oduro

**Affiliations:** Department of Population and Health, University of Cape Coast, Cape Coast, Ghana

**Keywords:** Older adults, Ghana, Social integration, Psycho-physical factors, Public health

## Abstract

**Background:**

Globally, older adults aged 60 years and over are outnumbering children under 5 and young people aged 15–24. Much evidence exists on the importance of high social integration and positive quality of life and health outcomes. However, evidence on how older adults are socially integrated in Ghanaian communities is limited. This study examined how self-reported well-being and quality of life (psychological and physical (psycho-physical) factors) predict the social integration of older adults in Ghana.

**Methods:**

A secondary analysis of longitudinal survey data of the 2014/15 Study on Global Ageing and Adult Health (SAGE Wave 2) conducted by the World Health Organization was applied. Older adults aged 60 years and older (*n* = 1,927) were included in this study. Multilevel logistic regression analyses were used to examine psycho-physical factors associated with high social integration among older adults. The output was reported as odds ratios (OR).

**Results:**

In general, social integration varied based on older adults’ demographic characteristics. Those in rural communities had lower odds of having high social integration (OR = 0.76, 95% CI = 0.56,1.03) when compared with older adults in urban areas. Having high physical and psychological well-being was associated with high social integration (OR = 1.90, 95% CI = 1.41, 2.57), (OR = 2.05, 95% CI = 1.56, 2.69). However, older adults with high levels of emotional and spiritual well-being were 9% and 7% (respectively) less likely to experience a high level of social integration (OR = 0.94, 95% CI = 0.71,1.24), (OR = 0.79, 95% CI = 0.60,1.04).

**Conclusion:**

The higher the level of self-reported psychological and physical well-being, the higher the social integration for older adults aged 60 years and over. However, the higher the level of self-reported emotional well-being and spiritual well-being, the less likely to have high social integration. Improved social integration or participation in society for older adults with high emotional and spiritual well-being is needed. The findings of this study highlight the need for policymakers and stakeholders to consider psycho-physical factors as an important public health tool and metric to encourage more research on the well-being of older adults in Ghana.

**Supplementary Information:**

The online version contains supplementary material available at 10.1186/s12877-024-04954-x.

## Introduction

There is a surge in global population ageing. Africa tops the list of regions that are experiencing the fastest growth in the population of older adults worldwide, (i.e., persons aged 60 years and older), followed by Latin America, the Caribbean, and Asia [1, 2]. Ghana, like many developing countries, e.g., Zimbabwe, Namibia, India, and Indonesia, will experience a rapid increase in the number of adults aged 60 years and older. The global population aged 60 years and over will reach 1.4 billion by 2030 and double to 2.1 billion by 2050, reflecting significant demographic shifts [1]. In Sub-Saharan Africa, the proportion of adults aged 60 and over is anticipated to reach 8.3% by 2050. Likewise, Ghana is expected to experience a rise to approximately 11.9% by 2050, although current statistics reveal that 13.1% of the population is already in this age group [2].

The surge in the older adult population, particularly in Ghana, implies quality health systems. Factors contributing to the ageing population in Ghana are decreased fertility and mortality rates, high life expectancy, technological advancements, and improved healthcare [3]. Despite the absence of a specific national policy for this age group, the exemption of older adults from health insurance premiums under Act 650 of Ghana’s health insurance scheme (NHIS) in 2003 appears pivotal to the rapid increase in their proportion [4].

Research into how older adults are socially integrated into communities is becoming increasingly important at a time when older adults 60 years and older are outnumbering children under 5 and young people aged 15–24 years worldwide [5]. High social integration refers to how well one is connected to their community and feels a sense of belonging, which is an important part of healthy ageing [6, 7]. Therefore, the extent to which older adults belong to social groups, visit friends in their neighbourhood, and participate in community meetings, plays a vital role in promoting high social integration and establishing age-friendly communities [7]. Despite the importance of social integration among older adults, studies on which psychological and physical (psycho-physical) factors predict high social integration are limited [1, 7–9].

The demographic shift toward an increasingly ageing population presents socio-economic challenges affecting the workforce, fiscal markets, and service demands in Ghana. These include education, housing, social protection, family structures, and intergenerational ties. Despite these challenges, the social integration of older adults receives limited attention in Ghanaian gerontological literature, necessitating a comprehensive examination of psycho-physical factors that predict their social integration [10–12].

Studies have shown the important role that social integration plays in promoting the overall well-being of older adults. Active involvement in social groups, socialising with friends, and engaging in diverse activities such as childcare have been identified as important contributors to improved health outcomes [13–15]. Additionally, income status, perceived health status, and family support have been recognised as influential determinants of social integration [16–18]. Moreover, cultural values play a role in preferences for specific social interactions [19]. Overall, social integration is a key factor in fostering healthy ageing, with strong associations observed with life satisfaction, perceived health, and physical well-being [20, 21]. Recent studies in Ghana show the impact of social protection initiatives, including the Livelihood Empowerment Against Poverty, pensions, cultural perceptions of witchcraft, and happiness, on the integration of older adults [22–25].

Although these studies offer valuable insights into critical factors influencing the social integration of older adults, they do not sufficiently address how psycho-physical factors contribute to the societal integration of older adults in Ghana. Consequently, there is limited understanding of the associations between psycho-physical factors and the social integration of older adults in Ghana. This study aimed to examine the association between psycho-physical factors, such as emotional, physical, psychological, and spiritual well-being, and the social integration of older adults in Ghana. By identifying which factors contribute to the high social integration of older adults, the results of this study may provide insight into the development of effective interventions and programs to better support the integration of older adults into Ghanaian society.

## Methods

This study used the comprehensive pooled data on the health and well-being of older adults in Ghana from the World Health Organisation 2014/15 Ghana Study on Global Ageing and Adult Health (SAGE) Survey Wave 2 [26]. SAGE Ghana is a longitudinal survey programme coordinated by the WHO’s multi-country studies unit and is a nationally representative study undertaken in six countries, including China, India, Mexico, the Russian Federation, and South Africa. The SAGE programme seeks to strengthen, gather, process, and manage data to understand the needs and challenges of older adults to inform policy, planning, and research (US National Institute on Aging [NIA] & WHO, 2014). SAGE Ghana is scheduled to collect data every four years up until 2018/19. Since this study is extracted from my Ph.D. thesis (2018–2022), and at the time SAGE 2018/19 data was not out, the 2014/15 (Wave 2) data was used for the analysis. The 2014/15 (Wave 2) SAGE collected some information on the well-being of older persons, including their emotional, physical, psychological, spiritual well-being, and social integration, in addition to socio-demographic and economic data.

SAGE surveys follow the standard procedures (i.e., sampling, questionnaire development, data collection, cleaning, coding, and analysis) which allow for cross-country comparison. The survey employs a stratified two-stage sampling technique. The initial stage involved stratification of the country by region and urban-rural location. Primary Sampling Units (PSUs), also referred to as Census Enumeration Areas from the 2010 Ghana Population and Housing Census, were allocated proportionately to the number of PSUs in each region by rural-urban location. PSUs were selected independently within each stratum and 214 PSUs were sampled. Households were selected in each PSU in the second stage using a systematic random sampling approach. All members aged 50 years and above were selected for interviews in the selected households. Overall, 3,575 respondents aged 50 years and above were interviewed. For the present study, only older adults 60 years and above (*n* = 1,927) with complete data were included in the analysis. Thus, older respondents below 60 years were excluded.

### Study variables

#### Outcome variables

Participants were categorised according to functional age brackets: 60–69 (young-old), 70–79 years (old-old), and 80 + years (oldest-old) [27, 28]. Social integration of the older adults was the outcome variable of interest. The outcome variable was measured by three variables as were used in the Ghana 2014/15 SAGE. In this study, social integration was conceptualised as the extent to which older adults participate in a range of social interactions including involvement in social relationships or activities (i.e., belonging to social groups, and visits to friends in the neighbourhood), and a sense of community (i.e., participation in community meetings). Given that the social integration of older adults is a latent indicator, SAGE Ghana wave 2 developed three series of questions to measure it, including “How often in the last 12 months, have you; 1) “socialised with co-workers outside of work?”, 2) “attended any group, club, society, union, or organisational meeting?”, and 3) “been in the home of someone who lives in a different neighbourhood than you do or had them in your home?”. Each question was rated on a 5-point Likert scale to reflect their self-rating, ranging from 1 = never, 2 = once or twice per year, 3 = once or twice per month, 4 = once or twice per week to 5 = daily [16, 27, 28].

To examine the predictors of the outcome variable of older adults, dummies were created for each of the predictors (*Socialised with co-workers, attended any group or society… meeting, had friends visit your home*) of social integration and were ranked using factor analysis. Thus, the factor analysis technique was used to create a composite variable to measure the overall social integration of older adults. The top 20.0% score was coded as “1” to represent high social integration, while “0” represents low social integration (Appendix I). This technique was adopted because it circumvents the problem of multicollinearity and assigns indicator weights based on the variations in the responses. The mathematical procedure in FA transforms correlated variables into fewer uncorrelated variables (factor scores). The factor scores are a linear combination of the original variables which are derived in decreasing order of importance with the first-factor score accounting for as much of the variability in the data as possible, and each succeeding factor accounting for as much of the remaining variability.

#### Independent variables

The independent variables considered as psycho-physical factors for the study were (1) emotional well-being, (2) physical well-being, (3) psychological well-being, and (4) spiritual well-being. Emotional well-being was measured as, in the last 30 days, how much of a problem did you have; (1) “with feeling sad, low, or depressed?” (2) “with worry or anxiety?” (3) “with feeling lonely?” And (4) “with feeling neglected?”. Physical well-being was measured using the functioning assessment scale. Questions used were, in the last 30 days, how much difficulty did you have; (1) “in bathing/washing your whole body?” (2) “with eating (including cutting up your food)?” and (3) “in vigorous activities (‘vigorous activities’ require hard physical effort and cause large increases in breathing or heart rate?” Psychological well-being was measured as, “Overall in the last 30 days, how much difficulty; 1) “did you have with concentrating or remembering things?” Each of the above questions was rated on a 5-point Likert scale to reflect their self-rating, ranging from 1 = None, 2 = Mild, 3 = Moderate, 4 = Severe to 5 = Extreme/Cannot do. 2) How often have you found that you could not cope with all the things that you had to do? 3) How often have you felt that you were unable to control the important things in your life? Each of these questions was rated on a 5-point Likert scale ranging from 1 = never, 2 = Almost never, 3 = Sometimes, 4 = Fairly often to 5 = Very often. Lastly, spiritual well-being was measured by 1) responses were 1 = No, none, 2 = Buddhism, 3 = Chinese traditional religion, 4 = Christianity (including roman catholic, protestant, orthodox, other), 5 = Hinduism, 6 = Others. “Do you belong to a religious denomination? 2)… attended religious services (not including weddings and funerals). This was also rated on a 5-point Likert scale to reflect their self-rating, ranging from 1 = never, 2 = once or twice per year, 3 = once or twice per month, 4 = once or twice per week to 5 = daily.

Also, dummies were created for each indicator of psycho-physical factors such as emotional, physical, psychological, and spiritual well-being. The first-factor score for each of the psycho-physical factors was used to represent the specific variable of well-being and was ranked using factor analysis. Thus, the factor analysis technique was used to create a composite variable to measure the overall psycho-physical factors. The top 20.0% score was coded as “1” to represent high emotional, physical, psychological, and spiritual well-being while “0” represents otherwise (Appendix II).

### Confounders

The confounders selected for the study included a range of demographic factors such as sex, age, marital status, education, ethnicity, residence, ecological zones, perceived health, work status, and income. These confounders were chosen based on evidence of their potential influence on the relationship between psycho-physical factors and social integration [16, 17, 22]. In the Ghanaian context, controlling for these factors is crucial due to their important impact on social integration. By accounting for these confounders, the study findings, allow for a more accurate understanding of the specific relationship between psycho-physical and social integration in Ghana. (Appendix III).

### Statistical analysis

Data was analysed using SPSS (version 26.0) and R studio (version 4.3.1). First, descriptive statistics were performed to describe older adults and their background characteristics. Second, cross-tabulations with chi-squared tests were used to examine the percentage distribution of the older adults with high social integration by the psycho-physical factors and the confounders. Lastly, multilevel binary logistic regression was used to examine the psycho-physical factors and confounders that were statistically significantly (*p* < 0.05) associated with high social integration of older adults.

### Multilevel binary logistic regression

Multilevel binary logistic regression was conducted to examine the psycho-physical factors associated with the probability of an older adult having high social integration, accounting for the confounders and the level of nesting of respondents at the community level. The approach prevented the possibility of underestimation or overestimation of model parameters due to the stratified nature of SAGE Ghana Wave 2 [29]. A two-level multilevel binary logistic regression model was fitted since the outcome variable of interest was dichotomous coded “1” if an older respondent had high social integration and “0” if otherwise and with individuals nested within communities. The implication of a statistically significant (*p* < 0.05) random variance at the community level is that the community in which the respondent resides influences his/her social integration. All the psycho-physical factors were retained in the model irrespective of their significance level, whilst only statistically significant confounders were retained in the model as suggested by Aitkin et al. [29, 30].

### Test for multicollinearity

To ensure that the model fitted was stable, a test of multicollinearity was conducted. The interval-by-interval Pearson’s R and the ordinal-by-ordinal Spearman correlation were used to examine the extent of correlations among categorical-by-categorical covariates. The results showed very low correlations among the variables, thus, low potential for multicollinearity.

### Model fitting procedure

A sequential modelling approach was used to examine the association between social integration and psycho-physical factors, accounting for the important background characteristics. Model 1 included the place of residence to account for some of the survey weights. Model 2 added the psycho-physical factors, while Model 3 included the background characteristics. A two-level (individuals nested within communities) multilevel binary logistic regression was used to examine the predictors of high social integration among older adults. Interpretation of all the model results was based on the final models (Table 3, Model 3).

The intraclass correlation coefficient (ICC) 2,1 was used to estimate the amount of variation in social integration of older adults that is attributable to differences in the community [31]. ICC values < 0.5 (poor variation), between 0.5 and 0.75 (moderate), between 0.75 and 0.9 (good), and > 0.90 (excellent) (Koo et al., 2016).

The Akaike Information Criterion (AIC) was used to determine the model of best fit (AIC). This AIC is used to select the model that reduces the negative likelihood relative to the number of parameters in the model [30]. Specifically, the model with the least expected information loss. Thus, the lower the AIC, the better the model and the closer it is to the unidentified population model [30]. SAGE collects data on the Subjective Well-being and Quality of Life of older adults including social integration and psycho-physical factors which improve the reliability of data about older adults in Ghana. Also, the study involved a large sample which confirm observations from previous studies, suggesting high validity of the results.

## Results

Of the 3, 575 older adults who participated in the study, 1,927 fulfilled the inclusion criteria and were included in the present analysis (Mean$\pm$SD 14.1$\pm$62.6 years; 43.2% male; 56.8% female). The participants predominantly comprised female (56.8%) aged between 60 and 80+ years (99.6%). Most of them were married (58.5%). Education levels vary, with roughly half (45.0%) having no formal education and a quarter (27.1%) having primary or junior high school education. Christianity was the dominant religion (72.8%), and the Akan (46.0%) and Mande-Busanga (23.6%) ethnic groups were well represented. Nearly half of the older adults (47.4%) engaged in full-time work and a majority reside in rural areas (60.9%). Most of the participants perceived their general health as good (68.2%). Apart from physical well-being (69.4%), respondents had high emotional (61.9%), psychological (54.9%), spiritual (80.2%) well-being, and social integration (71.6%). Complete information on participant characteristics is provided in Table 1.

**Table 1:** Participant Characteristics (*n* = 1927)

	Sample size	Percent (%)
Sex		
Male	833	43.2
Female	1094	56.8
Age		
60–69	1271	66
70–79	513	26.6
80+	143	7.4
Mean$\pm$SD		14.1$\pm$62.6
Marital status		
Not married	122	6.3
Married	1126	58.5
Separated/divorced	207	10.7
Widowed	472	24.5
Educational level		
No formal education	867	45.0
Primary or JHS	523	27.1
Secondary or higher	537	27.9
Religion		
Christianity	1402	72.8
Islam	355	18.4
No religion/Indigenous	170	8.8
Ethnicity		
Akan	886	46.0
Ewe	118	6.1
Ga-Adangbe	290	15.1
Mande-Busanga	454	23.6
Others	178	9.2
Work status		
Not working	372	19.3
Part-time work	642	33.3
Full-time work	913	47.4
Ecological Zones		
Savannah	418	21.7
Forest	956	49.6
Coastal	553	28.7
Place of residence		
Urban	753	39.1
Rural	1174	60.9
Income		
Low	448	23.3
High	1479	76.7
Perceived general health		
Poor	168	8.7
Moderate	444	23.0
Good	1315	68.2
*Psycho-physical factors*		
Emotional wellbeing		
Low	735	38.1
High	1192	61.9
Physical wellbeing		
Low	1337	69.4
High	590	30.6
Psychological wellbeing		
Low	870	45.1
High	1057	54.9
Spiritual wellbeing		
Low	382	19.8
High	1545	80.2
Social integration		
Low	548	28.4
High	1379	71.6

### Social integration, background characteristics, and psycho-physical factors

A high level of social integration differed by the demographic characteristics of older adults. 45% of the females had a high level of social integration. 41% of the old old (70–79 years) had a high level of social integration. Also, 47% who worked part-time and 46.3% who had perceived good general health status had a high level of social integration. Regarding the psycho-physical factors, respondents who had high levels of emotional (52.9%), physical (48.9%), psychological (56.1%), and spiritual (51.8%) well-being had a high level of social integration. Complete details are in Table 2.

**Table 2:** Background characteristics, psycho-physical factors by high social integration (*n* = 1927)

	Social integration	
Covariates	High	χ^2^_(p−value)_
	**%**	
*Background characteristics*		
Sex		26.166**
Male	33.9	
Female	45.4	
Age		6.461*
60–69 years	40.3	
70–79 years	41.4	
80 years or older	35.0	
Marital status		58.632**
Never married	32.8	
Married	43.9	
Cohabiting/Separated/divorced	47.1	
Widowed	24.8	
Educational level		60.472**
No formal education	30.9	
Primary/JHS	38.8	
Secondary/higher	51.8	
Religion		4.673
Christianity	39.7	
Islam	39.0	
No religion/Indigenous	31.2	
Ecological zones		11.992**
Savannah	31.6	
Forest	41.1	
Coastal	40.5	
Place of residence		4.410*
Urban	41.8	
Rural	37.0	
Ethnicity		32.704**
Akan	36.9	
Ewe	41.5	
Ga-Adangbe	50.7	
Mande-Busanga	31.6	
Others	46.1	
Main occupation		95.386**
Not working	17.2	
Part-time	47.0	
Full time	42.1	
Income		0.075
Low	39.4	
High	38.7	
Perceived general health		106.593**
Very bad	13.1	
Moderate	26.4	
Very good	46.3	
*Psycho-physical factors*		
Emotional wellbeing		97.231**
Low	30.3	
High	52.9	
Physical wellbeing		181.690**
Low	16.6	
High	48.9	
Psychological wellbeing		203.245**
Low	24.4	
High	56.1	
Spiritual wellbeing		33.699**
Low	35.7	
High	51.8	

χ^2^ = Chi square test, **p < 0.01, *p < 0.05

### Regression analysis of social integration and psycho-physical factors

Place of residence and physical and psychological well-being were found to be significant predictors of high social integration. Also, gender, work status, level of education, marital status, perceived general health, and income level had significant associations with the high social integration of older adults 60 years and older (Table 3).

When the place of residence was introduced in Model 1, the variance of the random effects at the community level reduced marginally (0.46%) and remained statistically significant at *p* < 0.05. This shows that significant differences exist between communities about older adults with high social integration, after accounting for the place of residence. When the psycho-physical factors (*i.e., emotional, physical, psychological, and spiritual well-being*) were included in model 2, the variance of the random effects at the community level declined by 0.57%. Still, the effect remained statistically significant at *p* < 0.05. Further indicating that significant differences exist between communities concerning high social integration among older adults, even after accounting for the place of residence and the psycho-physical factors. When the background characteristics were included in Model 3, the variance of the random effects at the community level increased by 0.72% and remained statistically significant at *p* < 0.05. This further indicates that the background factors increase the variability between communities. The statistically significant variance of the random effects at the community level after accounting for all the selected variables in the model indicates that the selected variables do not explain all the differences between communities about older adults with high social integration.

After controlling for important predictors in the model, the results indicate that older adults in the rural communities had lower odds (OR = 0.76, 95% CI = 0.56,1.03) of having high social integration when compared with their urban counterparts. Concerning the psycho-physical factors, older adults who had high levels of physical and psychological well-being were 19% and 20% more likely to be associated with a high level of social integration (OR = 1.90, 95% CI = 1.41, 2.57), (OR = 2.05, 95% CI = 1.56, 2.69) when compared with those who had low levels of physical and psychological well-being. However, older adults with high levels of emotional and spiritual well-being were 9% and 7% less likely to experience a high level of social integration (OR = 0.94, 95% CI = 0.71,1.24), (OR = 0.79, 95% CI = 0.60,1.04) when compared with those with a low level of emotional and spiritual well-being.

Being a female was 14% more likely to be associated with a high level of social integration (OR = 1.45, 95% CI = 1.13, 1.84) compared with being a male. Also, older adults who were ever married (married, separated/divorced, widowed) (OR = 2.35, 95% CI = 1.52, 3.64), (OR = 2.41, 95% CI = 1.44, 4.06), and (OR = 1.61, 95% CI = 0.99,2.63) respectively, had higher odds of high social integration when compared with older adults who were never married. Furthermore, older adults who engaged in full-time and part-time work were 23% and 22% more likely to be associated with a high level of social integration (OR = 2.30, 95% CI = 1.61, 3.29), (OR = 2.25, 95% CI = 1.59, 3.20) compared with those who were not working. Also, having a good perceived general health status was 26% more likely to be associated with the high rated level of social integration (OR = 2.69, 95% CI = 1.68, 4.31) when compared with having a poor perceived general health status. Details are presented in Table 3.

**Table 3:** Predictors of social integration of older adults

Community variable	Model 1	Model 2	Model 3
	OR [95% CI]	OR [95% CI]	OR [95% CI]
*Place of residence*			
Urban	1.00	1.00	1.00
Rural	0.84[0.64,1.11]	0.79[0.60,1.07]	0.76[0.56,1.03]
*Psycho-physical Factors*			
Emotional wellbeing			
Low		1.00	1.00
High		0.97[0.75,1.27]	0.94[0.71,1.24]
Physical wellbeing			
Low		1.00	1.00
High		2.30[1.73,3.05]**	1.90[1.41,2.57]**
Psychological wellbeing			
Low		1.00	1.00
High		2.28[1.76,2.94]**	2.05[1.56,2.69]**
Spiritual wellbeing			
Low		1.00	1.00
High		0.77[0.59,1.00]	0.79[0.60,1.04]
*Confounding factors*			
Sex			
Male			1.00
Female			1.45[1.13,1.84]*
Marital status			
Never married			1.00
Married			2.35[1.52,3.64] **
Separated/divorced			2.41[1.44,4.06] **
Widowed			1.61[0.99,2.63]
Work status			
Not working			1.00
Full time			2.25[1.59,3.20]**
Part time			2.30[1.61,3.29]**
General Health			
Very bad			1.00
Moderate			1.54[0.93,2.53]
Very good			2.69[1.68,4.31]**
Variance of the random effects [SE]			
Community	0.40[0.04]**	0.39[0.04]**	0.51[0.05] **
% ∆ in random effect	-0.46	-0.57	+ 0.72
Community			
Deviance	2378.5	2377.0	2255.6
AIC	2391.2	2390.5	2291.6

## Discussion

This study focused solely on high-level social integration because it is often associated with positive outcomes such as better mental health, increased social support, and overall well-being. Therefore, my interest was to understand the factors that contribute to this positive state. Also, examining the characteristics associated with high social integration gives insight into what facilitates good social connections among older adults. The major findings of this study are that older adults who had high levels of physical well-being and psychological well-being were 19% and 20% (respectively) more likely to have a high level of social integration (OR = 1.90, 95% CI = 1.41, 2.57), (OR = 2.05, 95% CI = 1.56, 2.69) when compared with those who had low levels of physical and psychological wellbeing. However, older adults with high levels of emotional and spiritual well-being were 9% and 7% less likely to experience a high level of social integration (OR = 0.94, 95% CI = 0.71,1.24), (OR = 0.79, 95% CI = 0.60,1.04) when compared with those with low levels of emotional and spiritual wellbeing.

Notably, a positive association emerged between a high level of psychological well-being and high social integration of older adults. This suggests an interconnectedness of positive cognitive function and social aspects of well-being. Findings align with existing research, indicating that positive mental health among older adults contributes to a more optimistic outlook on older adults’ lives, fostering a greater willingness to participate in social activities, socialising with friends, and childcare [32]. Equally, strong social ties and supportive social networks positively influence psychological well-being by providing companionship, emotional support, and a sense of belonging [20]. Conversely, evidence from a study by [33] showed high loneliness and low subjective well-being amongst Nepalese older adults. The findings of this study contribute to the dearth of literature on the important role that psychological well-being plays in the social integration of older adults in the Ghanaian context.

The study found a strong link between high physical well-being and high social integration of older adults. Social integration is crucial for healthy ageing, with evidence showing its strong associations with physical well-being. Previous studies indicated that social support and the perceived adequacy of social interaction contribute significantly to elderly individuals’ mental health and well-being [13, 20]. Additionally, good physical functioning is associated with physical activity and interaction, positively reflecting physical and functional well-being [14]. Maintaining good physical health allows older adults to participate in social networks, fostering strong social connections [14]. The study results add to the limited body of literature on gerontology and emphasise the influence of enhanced physical activity on the socially integrated lives of older adults in Ghana.

However, older adults with high levels of emotional well-being were less likely to experience a high level of social integration. Surprisingly, older adults who have higher emotional well-being may also have weaker social integration because of factors like a desire for private lives and self-sufficiency. This impression is influenced by a propensity for isolation and contentment with few social ties. Some cultural values in Ghana promote quality over quantity, where older persons are highly esteemed [34]. In addition, an older adult might be emotionally stable, but physical mobility may be limited by health issues, which might reduce their social integration levels and active involvement in social gatherings [35]. This finding complicates the oversimplified relationships between emotional well-being and social integration by emphasising the complex interactions between cultural context, personal preferences, and emotional health that influence older adults’ social integration in Ghana.

Also, older adults with a high level of spiritual well-being were less likely to be associated with a high level of social integration. Higher levels of spiritual well-being in older adults may encourage a more independent lifestyle through a preference for introspection and spiritual activities. Reduced involvement in social or community events could unintentionally result from this individual concentration on spirituality [36]. The prevailing cultural ideas linking spirituality to witchcraft may dissuade older adults from expressing their beliefs openly, leading them to keep their beliefs to themselves and participate less in social or group activities [36]. Furthermore, those who have a high level of spiritual well-being sometimes struggle with health issues that prevent them from actively participating in social gatherings.

The results from the study show that older adults in rural communities had lower odds of having high social integration. Compared to urban areas, older adults have fewer opportunities for social contact in rural areas due to a lack of social resources including community centres and recreational facilities [37]. Traditional traditions in some Ghanaian rural communities may value intimate family relationships over larger social networks, which could limit older folks’ ability to interact socially with others outside of their immediate families.

Furthermore, the findings of this study show that females had higher social integration than males. Due to their important function in both family and community life, older women frequently take on caregiving tasks, which leads to the development of close social bonds [22]. Conventional responsibilities, like taking care of family members, support larger social networks and exchanges. Older women actively engage in local initiatives, religious groups, and community activities in many traditional societies, which opens doors for social integration through community participation [22].

Being ever married (married, separated/divorced, widowed) had higher odds of high social integration. A core family unit is established by marriage, and spouses are essential providers of emotional support [22]. Ever married people may find grounds for strong social integration inside their own family, particularly in countries like Ghana. Social expectations associated with marriage include fulfilling community norms, interacting with one’s spouse’s groups, and participating in social events, all of which improve social integration [17].

Also, older adults who ever worked were more likely to experience a high level of social integration. Employment serves as a platform for building social networks, offering opportunities for older adults to establish strong connections with colleagues. Whether in full-time or part-time roles, the workplace fosters shared experiences and common goals, creating a foundation for ongoing social interactions even after retirement [16]. Retirees often maintain social connections through social clubs, or associations tied to their former workplaces, enhancing their social integration.

Perceived good health status was more likely to be associated with a high level of social integration. When older adults perceive their health to be good, they commonly have improved mobility and actively participate in social activities, community events, and social interactions [20]. This sense of good health encourages self-assurance, allowing older adults to establish and maintain relationships and increase their level of social integration. It encourages involvement in social groups and community projects, forming relationships, and promoting a feeling of community for older adults.

### Strengths and limitations

One strength of this study was the large and nationally representative data sets from the SAGE 2014/15 Wave 2. Also, only variables that showed significant association with social integration were included in the multilevel logistic regression analysis. This supports the robustness of the models and reinforces the reliability and replicability of the findings of this study. Regarding the limitations, since a cross-sectional design was used, some caution should be applied to the interpretation of the study results. This is because the study examined association and therefore it is difficult to establish the causality of psycho-physical factors in the social integration of older adults in Ghana. Since variables in this study were self-rated by older adults, there is a tendency that respondents to rate their responses in a way considered favourable by others. As such, there is the possibility of recall bias as well as social group desirability bias. Also, because of the self-rated nature of social integration variables, there is the possibility of underreported issues regarding social integration. Further, leaving out low-level social integration for a narrow focus on high-level social integration results in a lack of understanding of the needs and experiences of those with lower social integration. This method could produce skewed findings on the elements of social integration and how it affects a range of demographic groups. Also, opportunities for focused interventions and assistance for vulnerable older adults are obscured when low-level social integration is neglected.

### Conclusion and policy implications

This study examined the psycho-physical factors and social integration of older adults in Ghana. The study has shown that the higher the level of self-reported psychological well-being and physical well-being, the higher the social integration for older adults aged 60 years and over. On the contrary, the higher the level of self-reported emotional well-being and spiritual well-being, the less likely to have high social integration. Thus, high emotional well-being and spiritual well-being among older adults suggest that they require more participation in society/high social integration. In comparison, high physical well-being and psychological well-being are associated with improved social integration among older adults aged 60 years and older in Ghana. Therefore, social integration in Ghana is influenced by the psycho-physical factors of older adults. Understanding the factors associated with high social integration can inform interventions and policies aimed at promoting social connectedness and well-being among older adults. Also, by identifying demographic characteristics and psycho-physical factors that are conducive to high social integration, interventions can be targeted more effectively. This has implications for community-wide initiatives by the policymakers and stakeholders to involve older adults in community-based activities such that older adults could serve as advisory bodies for younger adults and children which could enhance their emotional health. It also implies that religious leaders need to engage older adults in their religious activities and services to improve interpersonal relations among older and younger adults (i.e., intergenerational ties) while ensuring their spiritual well-being. While these specifically improve the emotional, and spiritual well-being and social integration of older adults, they would have positive effects on their physical and psychological wellbeing. Thus, highlighting the important role played by the psycho-physical factors in the social integration of older adults in Ghana.

### Electronic supplementary material

Below is the link to the electronic supplementary material.

Supplementary Material 1

## Data Availability

The datasets analysed during the current study are available at https://apps.who.int/healthinfo/systems/surveydata/index.php/access_licensed/track/4087.

## Abbreviations

AIC: Akaike Information Criterion
CI: Confidence Interval
GSS: Ghana Statistical Service
ICC: Intraclass Correlation Coefficient
NHIS: National Health Insurance Scheme
NIA: National Institute on Aging
OR: Odds Ratio
SAGE: Study on Global Ageing and Adult Health
SE: Standard Error
UN: United Nations
US: United States
WHO: World Health Organisation
WPAR: World Population Ageing Report
