# Supplementary material for: Psychological and physical factors related to social integration of older adults in Ghana
Source: BMC Geriatr. 2024 Apr 23;24:363. doi: 10.1186/s12877-024-04954-x (PMC11040942; doi:10.1186/s12877-024-04954-x)
Supplement: Supplementary file 1 — Supplementary Material 1 [file 12877_2024_4954_MOESM1_ESM.docx]

Appendices

Principal Component Analysis was conducted to identify the principal components that capture the most variance in social integration. The factor loadings of each variable (*i.e., Socialised with co-workers, attended any group, society… meeting, and had friends visit your home*) were examined to understand their contribution to social integration.

**Scoring System**

Weights were assigned to the principal components based on the proportion of variance explained in social integration. For example, the first principal components used explained approximately 80% of the variance in social integration for each factor. Therefore, the weight assigned was 8 for each individual variable.

The distribution of cut-off points for social integration was calculated using the fourth quintile of the indicator variables. These were ranked in the SPSS statistic application as follows; values above 25 were placed in the “very high” category, values between 23.8 and 24.2 were in the high social integration category, moderate social integration consisted of values between 21.6 and 22.5, and the low social integration category included values under 20.0 (Martinez-Martinezet al., 2016; Roser, 2014; UNDP, 2012). Therefore, using the cut-off point, this study considered the values above 21 as high social integration. Thus, the top 40% was used to measure high social integration on a scale ranging from 0 to 50.

**Interpretation**

A higher index score indicates higher levels of social integration. Based on the scale used, a score above 40% indicated high social integration, while a score below 20% suggested low social integration.

Appendix I

**Social Integration, Indicators, and Factor Loadings**

| **Outcome variable** | **Indicator** | **Factor loading** |
| --- | --- | --- |
| Social integration | 1. Socialised with co-workers | 0.790 |
|  | 1. Attended any group, society… meeting | 0.822 |
|  | 1. Had friends visit your home | 0.774 |

The same technique, as used in creating a single index for social integration was applied to create a composite index for each psycho-physical factor such as emotional well-being, physical well-being, psychological well-being and spiritual well-being.

Appendix II

**Psycho-physical Factors, Indicators, and Factor Loadings**

| **Domain** | **Indicator** | **Factor loadings** |
| --- | --- | --- |
| Emotional wellbeing | Not depressed | 0.813 |
|  | Not worried | 0.761 |
|  | Do not feel lonely | 0.794 |
|  | Do not feel neglected | 0.774 |
| Physical wellbeing | Able to bath/wash whole body | 0.848 |
|  | Able to eat and cut up food | 0.816 |
|  | Able to do moderate/vigorous exercise | 0.704 |
| Psychological wellbeing | Able remember things | 0.924 |
|  | Able to concentrate on things I do | 0.907 |
|  | Able to cope in difficult times | 0.850 |
|  | Have control over what I do | 0.741 |
| Spiritual wellbeing | Belong to a religion/church | 0.929 |
|  | Participate in religious services | 0.691 |

Appendix III

**Confounding Factors**

| **Variables** | **Measurement** |
| --- | --- |
| Sex | 0=Male |
|  | 1=Female |
| Age | 0=60-69 [young old] |
|  | 1=70-79 [old old] |
|  | 2=80+ [oldest old] |
| Marital status | 0=Not married |
|  | 1=Married |
|  | 2=Cohabiting/separate/divorced |
|  | 3=Widowed |
| Educational level | 0=No formal education |
|  | 1=Primary/JHS |
|  | 2=Secondary/Higher |
| Ethnicity | 0=Akan |
|  | 1=Ewe |
|  | 2=Ga-Adangbe |
|  | 3=Mande-Busanga |
|  | 4=Others |
| Place of residence | 0=Urban |
|  | 1=Rural |
| Ecological zones | 0=Savannah |
|  | 1=Forest |
|  | 2=Coastal |
| Perceived general health | 0=Very bad |
|  | 1=Moderate |
|  | 2=Very good |
| Work status | 0=Not working |
|  | 1=Full time |
|  | 2=Part time |
| Income | 0=Low income |
|  | 1=High income |
